# Overexpression of RPS27a contributes to enhanced chemoresistance of CML cells to imatinib by the transactivated STAT3

**DOI:** 10.18632/oncotarget.7888

**Published:** 2016-03-03

**Authors:** Houcai Wang, Bingqian Xie, Yuanyuan Kong, Yi Tao, Guang Yang, Minjie Gao, Hongwei Xu, Fenghuang Zhan, Jumei Shi, Yiwen Zhang, Xiaosong Wu

**Affiliations:** ^1^ Department of Hematology, Shanghai Tenth People's Hospital, Tongji University School of Medicine, Shanghai, China; ^2^ Department of Internal Medicine, University of Iowa, Carver College of Medicine, Iowa, USA

**Keywords:** STAT3, RPS27a, imatinib, apoptosis, CML

## Abstract

STAT3 plays a pivotal role in the hematopoietic system, which constitutively activated by BCR–ABL via JAK and Erk/MAP-kinase pathways. Phospho-STAT3 was overexpressed in imatinib-resistant CML patients as relative to imatinib responsive ones. By activation of the STAT3 pathway, BCR–ABL can promote cell cycling, and inhibit differentiation and apoptosis. Ribosomal protein S27a (RPS27a) performs extra-ribosomal functions besides imparting a role in ribosome biogenesis and post-translational modifications of proteins. RPS27a can promote proliferation, regulate cell cycle progression and inhibit apoptosis of leukemia cells. However, the relationship between STAT3 and RPS27a has not been reported. In this study, we detected a significantly increased expression of STAT3 and RPS27a in bone marrow samples from CML-AP/BP patients compared with those from CML-CP. In addition, we also demonstrated that it was a positive correlation between the level of STAT3 and that of RPS27a. Imatinib-resistant K562/G01 cells expressed significantly higher levels of STAT3 and RPS27a compared with those of K562 cells. RPS27a could be transactivated by p-STAT3 through the specific p-STAT3-binding site located nt −633 to −625 and −486 to −478 of the RPS27a gene promoter in a dose-dependent manner. The transactivated RPS27a could decrease the percentage of apoptotic CML cells induced by imatinib. And the effect of STAT3 overexpression could be counteracted by the p-STAT3 inhibitor WP1066 or RPS27a knockdown. These results suggest that drugs targeting STAT3/p-STAT3/RPS27a combining with TKI might represent a novel therapy strategy in patients with TKI-resistant CML.

## INTRODUCTION

Chronic myelogenous leukemia (CML) is a common hematologic malignancy, characterized by the formation of Philadelphia (Ph) chromosome and BCR-ABL fusion gene [1]. As a constitutively active tyrosine kinase, BCR-ABL protein gives rise to uncontrolled growth of myeloid cells in the bone marrow through a series of downstream pathways [2]. The tyrosine kinase inhibitor (TKI) imatinib is a specific molecular target-drug for the treatment of Ph chromosome-positive CML [3–6]. Although it is considered as one of the most effective drugs and the first-line treatment for CML, resistance to imatinib seems unavoidable and occurs frequently during its clinical application. Before the era of TKIs, CML patients in chronic phase (CML-CP) progressed to a more accelerated phase (CML-AP) after a median interval of about 5 years. CML-AP patients might still respond to treatment for months or years, but eventually developed a very aggressive blast phase (CML-BP), after which the median survival was about 6 months. Some patients progressed directly to CML-BP without an intermediate phase of acceleration. CML-BP is associated with dramatic changes in the leukemia cell phenotype: enhanced “stemness”, uncontrolled proliferation and invasion, abrogated differentiation, and early resistance to TKIs [7, 8]. Previous researches demonstrated that response to imatinib in patients with advanced CML was less prominent than that in CML-CP [9, 10]. At present, the molecular mechanisms responsible for these extensive changes are still uncertain; most likely, they involve activation of oncogenic factors, inactivation of tumor suppressors, or both. And abnormal expression of some other genes (P-glycoprotein (p-gp), AXL, heat-shock protein 70 (Hsp-70), STAT3, STAT5, Cancerous inhibitor of protein phosphatase 2A (CIP2A), B cell-specific MLV inte-gration site-1 (BMI-1), ATP-binding cassette sub-family G member 2 (ABCG2) were highly expressed and SHP-1, Cyclin-Dependent Kinase Inhibitor 3 (CDKI3), Raf Kinase Inhibitor Protein (RKIP), NOTCH2 were lowly expressed) or alternative signaling pathways activation may also contribute to imatinib resistance [11–26].

Ribosomal protein S27a (RPS27a) is one of two (the other one is L40) ribosomal proteins naturally synthesized as an ubiquitin (Ub) C-terminal extension protein [27, 28]. Besides imparting a role in ribosome biogenesis and post-translational modifications of proteins, RPS27a could perform extra-ribosomal functions [29, 30]. In previous researches, we found that the expression level of RPS27a was significantly higher in patients with CML-AP/BP than that in patients with CML-CP. The expression level of RPS27a was high in K562 cells and even higher in imatinib resistant K562/G01 cells. The ablation of RPS27a expression could inhibit the proliferation, induce cell cycle arrest and potentiate the effect of imatinib on apoptosis of K562 and K562/G01 cells partially through Raf/MEK/ERK, P21 and BCL-2 signaling pathways [31]. As the level of RPS27a expression was associated with clinical stages in CML patients in our study, it was speculated that RPS27a might be involved in the transformation of CML-CP to CML-AP/BP and implicated in the response to imatinib treatment.

To explore the mechanism of RPS27a up-regulation in the transition from CML-CP to CML-AP/BP, We hypothesized that some highly expressed proteins (P-gp, AXL, Hsp-70, STAT3, STAT5, CIP2A, BMI-1 and ABCG2) in imatinib resistant CML cells contributed to the up-regulation of RPS27a gene [13–19, 21, 22, 32]. The transcriptional regulation of RPS27a was investigated using the luciferase reporter assay after transiently transfecting cells with some common transcription factors such as P-gp, AXL, Hsp-70, STAT3, STAT5, CIP2A, BMI-1 and ABCG2. There was a significant (about twelve-fold) increase in the FL-RPS27a reporter gene activity in the presence of STAT3. It implied that STAT3 might transactivate RPS27a gene. And we detected the level of STAT3 and RPS27a in bone marrow samples from CML-AP/BP and CML-CP Patients. We found that STAT3 was up-regulated in the transition from CML-CP to CML-AP/BP and there was a positive correlation between the expression level of STAT3 and that of RPS27a. And imatinib resistant K562/G01 cells expressed significantly higher levels of STAT3 and RPS27a compared with those of K562 cells. STAT3 played a pivotal role in the hematopoietic system. BCR-ABL could induce activation of STAT3 via two different pathways, i.e. JAK and Erk/MAP-kinase [32, 33]. And the level of p-STAT3 was found to be higher in imatinib resistant CML patients than in imatinib responsive ones. In the study, we explored the possible relationship of STAT3 and RPS27a and their roles in CML cells. We found that RPS27a could be transactivated by p-STAT3 through the specific p-STAT3-binding site located nt −633 to −625 and nt −486 to −478 in the RPS27a gene promoter in a dose-dependent manner. Importantly, it was reported for the first time, that RPS27a transactivated by p-STAT3 could inhibit the effect of imatinib on apoptosis of K562 cells.

## RESULTS

### STAT3 is a key activator of the RPS27a promoter

**Figure 1:**
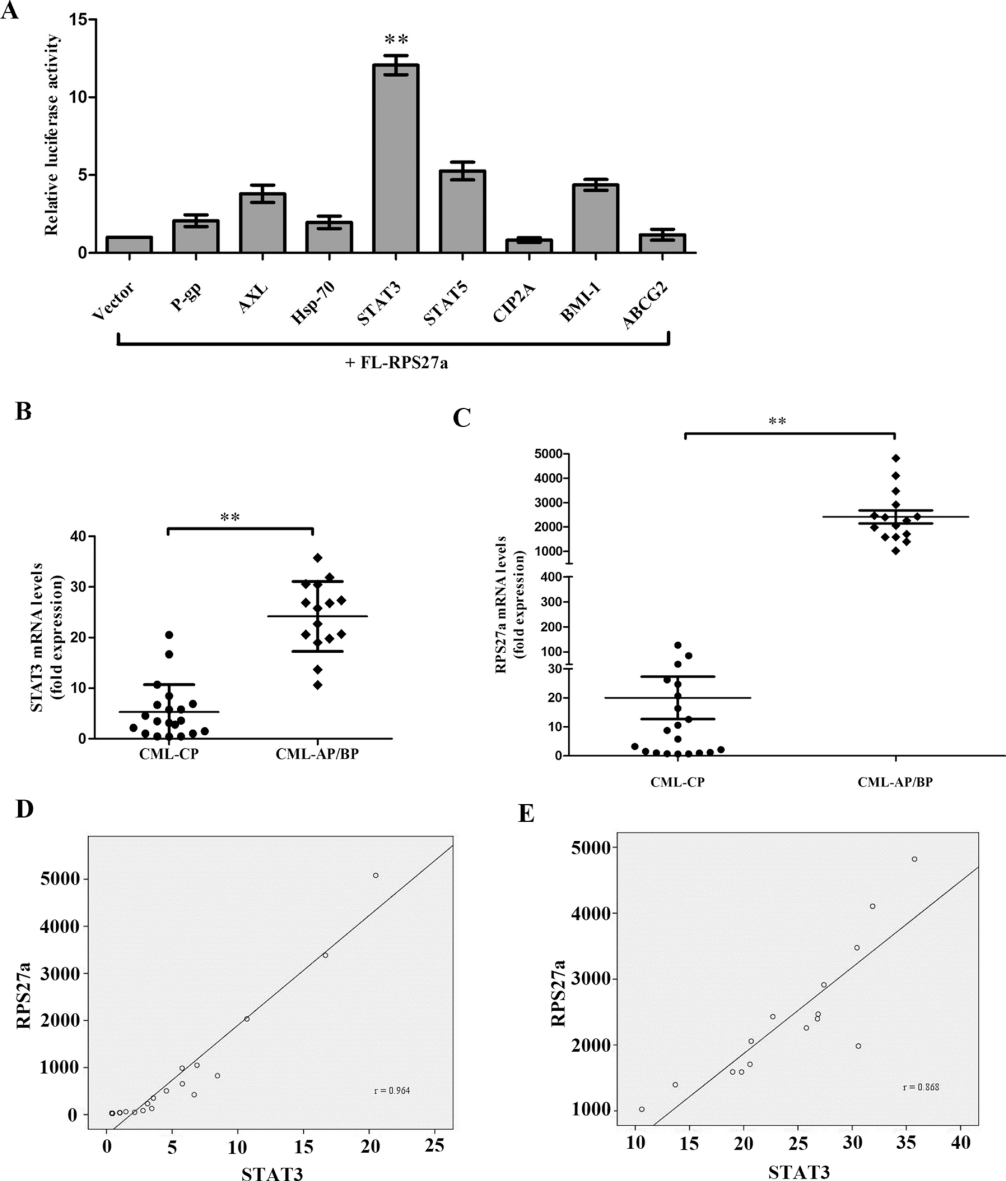
Relationship of STAT3 and RPS27a and their expression in CML patients (**A**) The DNA sequence of 1.6-kb full-length human RPS27a gene was cloned into luciferase reporter constructs. HEK-293 cells were transfected with FL-RPS27 a along with either vector control or express ion plasmi ds for P-gp, AXL, Hsp-70, STAT3, STAT5, CIP2A, BMI-1 and ABCG2. Cells were harvested after 48 h and total cell lysates were used for the luciferase assay. (**B** and **C**) Differential expression of STAT3 and RPS27a mRNA in patients with CML-CP or CML-AP/BP was illustrated in scatter plots. (**D** and **E**) The correlation of STAT3 and RPS27a was analyzed by Spearman correlation test. Columns and bars represent mean from 3 parallel experiments and SD, respectively. *P < 0.05; **P < 0.01.

To explore the mechanism of RPS27a up-regulation in the transition from CML-CP to CML-AP/BP, We hypothesized that some highly expressed proteins (P- gp, AXL, Hsp-70, STAT3, STAT5, CIP2A, BMI-1 and ABCG2) in imatinib resistant CML cells contributed to the up-regulation of RPS27a gene. The DNA sequence of 1.6-kb full-length human RPS27a gene (FL-RPS27a) was cloned into luciferase reporter constructs. The transcriptional regulation of RPS27a was investigated using the luciferase reporter assay after transiently transfecting cells with pCMV-P-gp, pCMV-AXL, pCMV-Hsp-70, pCMV-STAT3, pCMV-STAT5, pCMV-CIP2A, pCMV-BMI-1 and pCMV-ABCG2. There was a significant (about ten-fold) increase in the FL-RPS27a reporter gene activity in the presence of pCMV-STAT3 (Figure 1A). It implied that STAT3 was a key activator of the RPS27a promoter.

### STAT3 and RPS27a are highly expressed in CML-AP/BP, and K562/G01 cells; and there is a positive correlation between STAT3 and RPS27a

**Figure 2:**
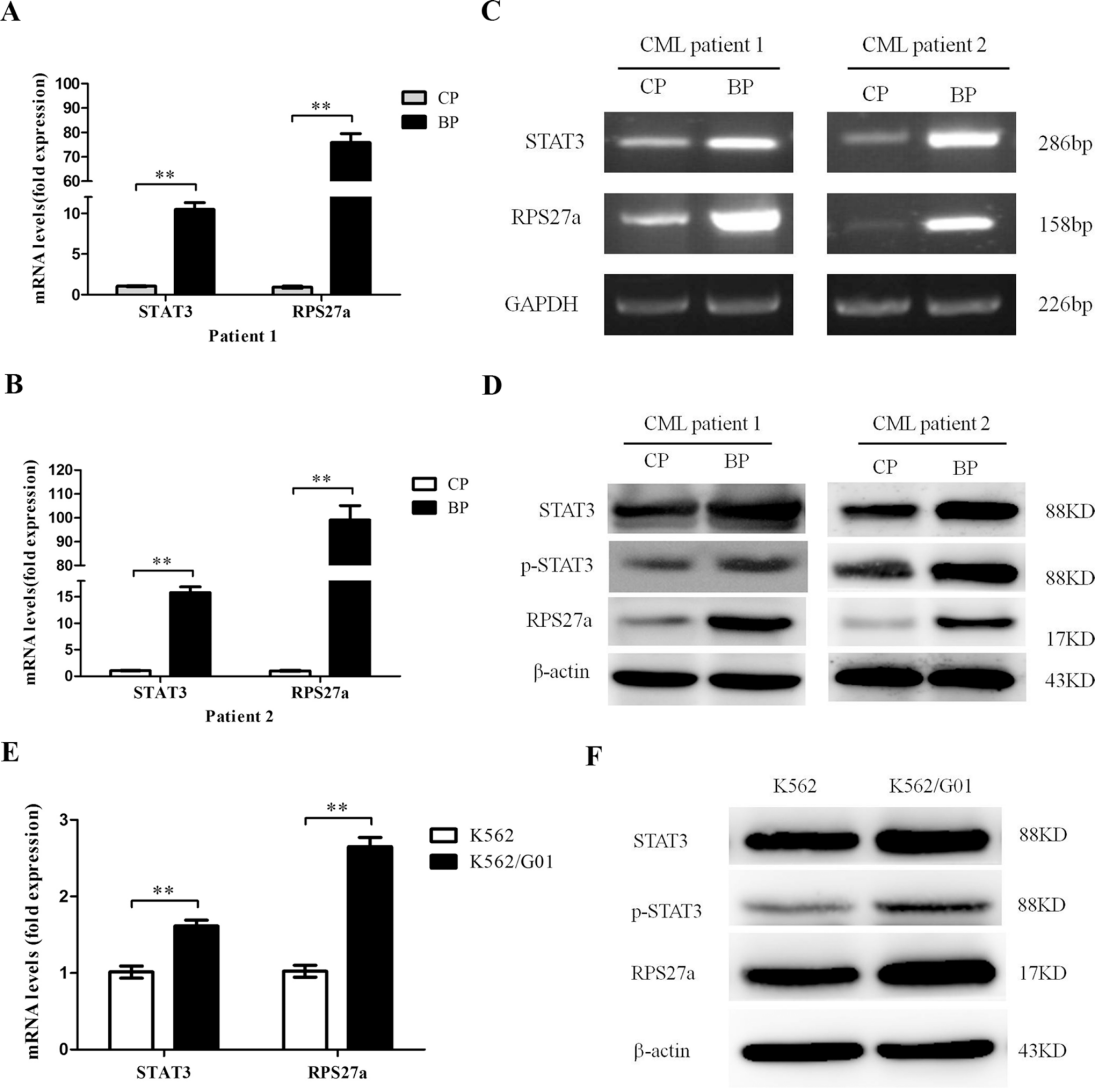
Expression of STAT3 and RPS27a in different phases of two CML patients and K562, K562/G01 cells (**A** and **B**) Relative mRNA expression of STAT3 and RPS27a was assessed in different phases of two CML patients by qRT-PCR. (**C**and **D**) Lysates of bone marrow cells from two CML patients in different phases were analyzed for STAT3, p-STAT3 and RPS27a protein by Western blot. (**E**) The qRT-PCR was performed to determine the mRNA levels of STAT3 and RPS27a in K562 and K562/G01cells. (**F**) The Western blot was performed to determine the protein levels of STAT3, p-STAT3 and RPS27a in K562 and K562/G01cells. Columns and bars represent mean from 3 parallel experiments and SD, respectively. *P < 0.05; **P < 0.01.

To explore the possible role of STAT3 and RPS27a in CML cells, we first investigated the expression levels of STAT3 and RPS27a in CML-CP and CML-AP/BP. Relative quantification using qRT-PCR revealed a striking increase of STAT3 and RPS27a mRNA expression in bone marrow samples from CML-AP/BP patients than that from CML-CP patients (Figure 1B and 1C). And there was a positive correlation between the expression level of STAT3 and RPS27a (Figure 1D and 1E). Then we detected the expression level of STAT3 and RPS27a in bone marrow samples from respective CP and BP of two CML patients. We found that STAT3 and RPS27a were simultaneously up-regulated in the transition from CP to BP of the two CML patients (Figure 2A, 2B, 2C and 2D). We also compared the expression levels of STAT3 and RPS27a in K562 with those of K562/G01 cells in order to explore whether STAT3 and RPS27a played a role in the responsiveness of cells to imatinib. It was found that K562/G01 cells expressed significantly higher levels of STAT3, p-STAT3 and RPS27a compared with those of K562 cells (Figure 2E and 2F). It was inferred that STAT3 and RPS27a might be involved in the transition from CP to BP of CML or the responsiveness of CML cells to imatinib treatment.

### RPS27a can be up-regulated by P-STAT3 in HEK293T cells

**Figure 3:**
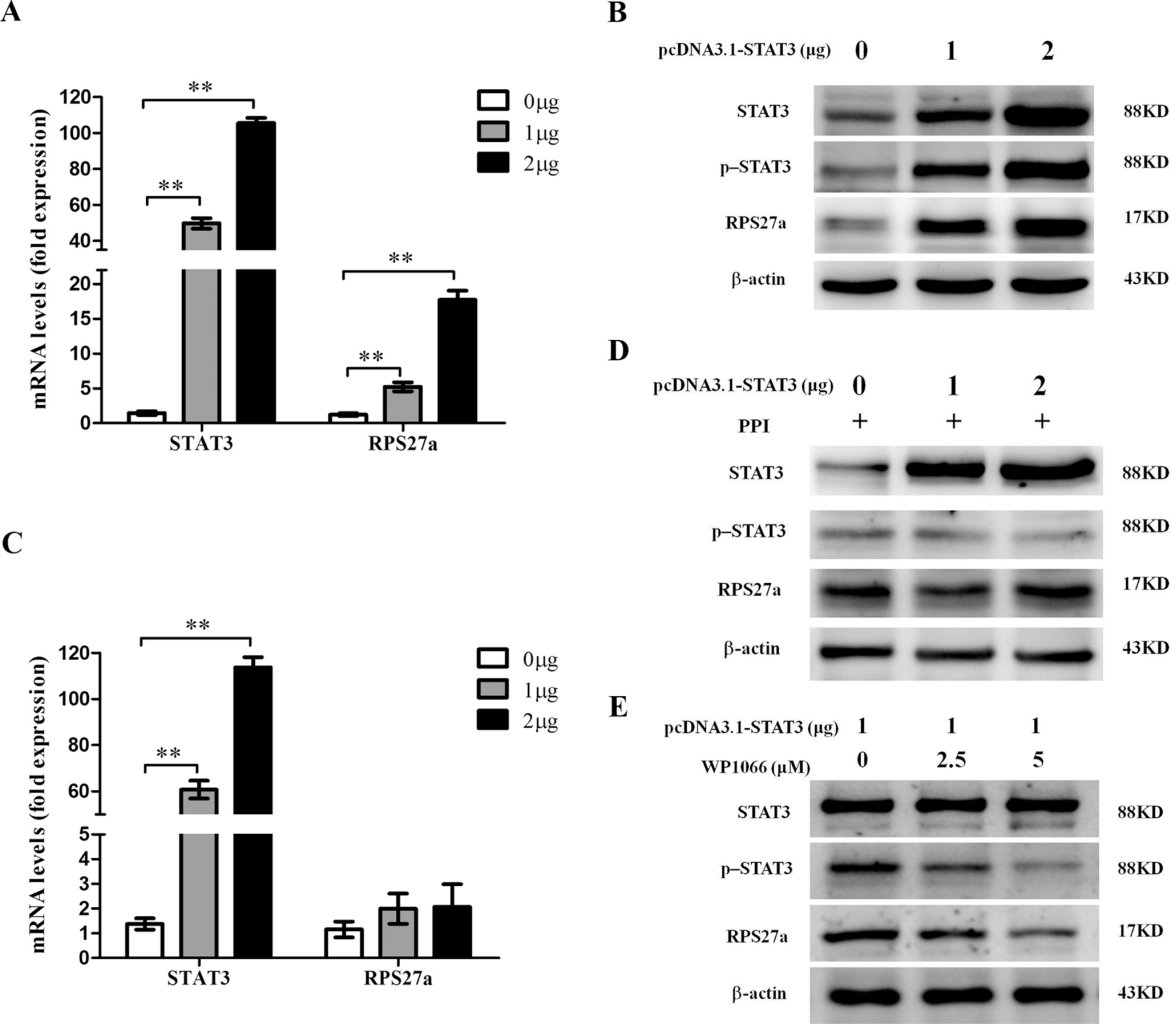
Upregulation of RPS27a by p-STAT3 in HEK293T cells (**A**) Relative RPS27a mRNA expression was assessed in HEK293T cells transfected with different dose of pcDNA3.1-STAT3 plasmid by qRT-PCR. (**B**) Lysates of HEK293T cells transfected with different dose of pcDNA3.1-STAT3 plasmid were analyzed for STAT3, p-STAT3 and RPS27a protein by Western blot. (**C**) Relative RPS27a mRNA expression was assessed in HEK293T cells transfected with different dose of pcDNA3.1-STAT3 plasmid and incubated with protein phosphatase inhibitor (PPI) by qRT-PCR. (**D**) Lysates of HEK293T cells transfected with different dose of pcDNA3.1-STAT3 plasmid and incubated with PPI were analyzed for STAT3, p-STAT3 and RPS27a protein by Western blot. (**E**) Lysates of HEK293T cells transfected with the same dose of pcDNA3.1-STAT3 plasmid and incubated with different dose of P-STAT3 inhibitor WP1066 were analyzed for STAT3, p-STAT3 and RPS27a protein by Western blot. Columns and bars represent mean from 3 parallel experiments and SD, respectively. *P < 0.05; **P < 0.01.

To explore the possible relationship of STAT3 and RPS27a, we investigated the changes of p-STAT3 and RPS27a expression after STAT3 was over-expressed in HEK293T cells. Relative quantification using qRT-PCR revealed a striking increase of RPS27a mRNA expression as STAT3 was overexpressed in HEK293T cells (Figure 3A). The overexpression of STAT3 upregulated levels of p-STAT3 protein and RPS27a protein (Figure 3B). When the phosphorylation of STAT3 was inhibited, the up-regulation of RPS27a didn't occur in HEK293T cells (Figure 3C and 3D). WP1066 is one of p-STAT3 inhibitors. The level of RPS27a was down-regulated as the p-STAT3 was inhibited by WP1066 gradually. The above data indicated that the up-regulation of RPS27a was dependent on the phosphorylation of STAT3.

### RPS27a can be transactivated by P-STAT3

**Figure 4:**
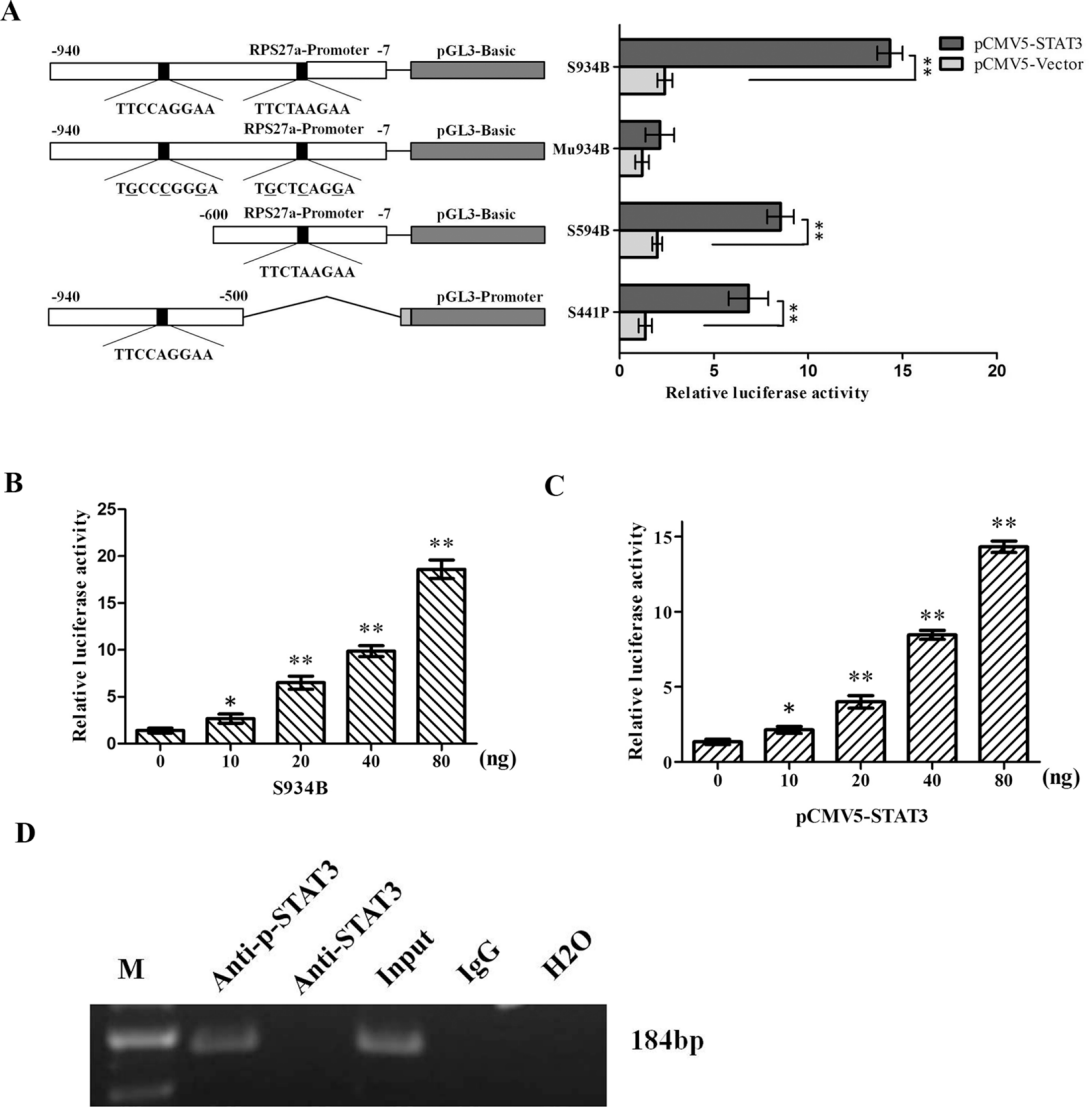
Effects of p-STAT3 on the transcriptional activity of RPS27a promoter (**A**) The constructions of various truncated RPS27a promoter-luciferase reporter plasmids and the transcriptional activity analysis. Different RPS27a promoter DNA fragments were fused to a luciferase reporter vector pGL3-promoter or pGL3-basic. The putative p-STAT3-binding sites were presented by black spots. Mu934B has six bases (underlined) different from those in S934B. P: pGL3-promoter, B: pGL3-basic. Each of the constructs was co-transfected with pCMV5-STAT3 into HEK293 cells. At 48 h after transfection, a six-fold increase in luciferase activity of S934B was observed in cells transfected with pCMV5-STAT3 at a dose of 80 ng. No changes were observed in Mu934B groups. (**B** and **C**) The luciferase transcriptional activity of S934B was activated by p-STAT3 in a dose-dependant manner. (**D**) Chromatin immunoprecipitation analysis of p-STAT3 binding to the RPS27a promoter in k562/G01 cells. The blot is representative of three experiments. The promoter fragment of 184 bp containing the putative p-STAT3-binding site at nt −633 to −625 and nt −486 to −478 of RPS27a was successfully amplified, both from the input DNA and the chromatin immunoprecipitated by anti-p-STAT3 antibody, whereas no amplified product was obtained in the anti-STAT3 antibody group, the immunoglobulin G isotype control group and H2O blank group. Columns and bars represent mean from 3 parallel experiments and SD, respectively. *P < 0.05; **P < 0.01.

As shown in the above study, the endogenous expression of RPS27a could be up-regulated by p-STAT3, and on the other hand, there are putative p-STAT3-binding sites within the promoter region of RPS27a gene; there might be possible links between transactivation of RPS27a gene and p-STAT3 binding to its promoter. To this end, we generated a serial of RPS27a luciferase reporter gene constructs to analyze the trans-active effect of p-STAT3 on RPS27a. Of them, S934B containing two putative p-STAT3 protein-binding sites (nt −633 to −625 TTCCAGGAA and −486 to −478 TTCTAAGAA) and S594B containing one putative p-STAT3 protein-binding sites (nt −486 to −478 TTCTAAGAA) were fused to luciferase reporter vector pGL3-basic; whereas S441P containing one putative p-STAT3-binding site (nt −633 to −625 TTCCAGGAA) to pGL3-promoter vector (Figure 4A).

Each of these luciferase reporter gene constructs was co-transfected with pCMV5-STAT3 into HEK293 cells, respectively. The luciferase activity was analyzed by luminometer at 48 h after transfection. To demonstrate this ‘site-specificity’, Mu934B with mutated p-STAT3-binding site (nt −633 to −625 TGCCCGGGA and −486 to −478 TGCTCAGGA) was co-transfected with pCMV5-STAT3 into HEK293 cells. As shown in Figure 4A, the luciferase activity of S934B, S594B and S441B were activated by p-STAT3. And the luciferase activity of S934B were the strongest. While the luciferase activity of Mu934B couldn't be activated by p-STAT3. It indicated that the element required for p-STAT3 transactivation activity was mainly located nt −633 to −625 and nt −486 to −478 upstream of the RPS27a transcription start site. Moreover, p-STAT3 transactivated the RPS27a promoter in a dose-dependent manner. S934B luciferase activity was observed to be up-regulated as the dose of S934B increased. (Figure 4B) and a 6-fold increase in S934B luciferase activity was observed in pCMV5-STAT3 group at a dose of 80 ng. (Figure 4C). These results showed that p-STAT3 could transactivate RPS27a in a dose-dependent manner. To further confirm the specific binding of p-STAT3 to RPS27a promoter at nt −633 to −625 and nt −486 to −478, chromatin immunoprecipitation assays were performed using K562/G01 cells. As shown in Figure 4D, the fragment between nt −658 and −469 containing the putative p-STAT3-binding site at nt −633 to −625 and −486 to −478 of RPS27a was successfully amplified, both from the input DNA and the chromatin immunoprecipitated by anti-p-STAT3 antibody, whereas no amplified product was obtained in the anti-STAT3 antibody group, the immunoglobulin G isotype control group and the H2O blank control group. The chromatin immunoprecipitation data demonstrated the *in vivo*-specific binding of p-STAT3 to promoter of the RPS27a gene.

### Transactivation of RPS27a by P-STAT3, regulates chemosensitivity of CML cells to imatinib

**Figure 5:**
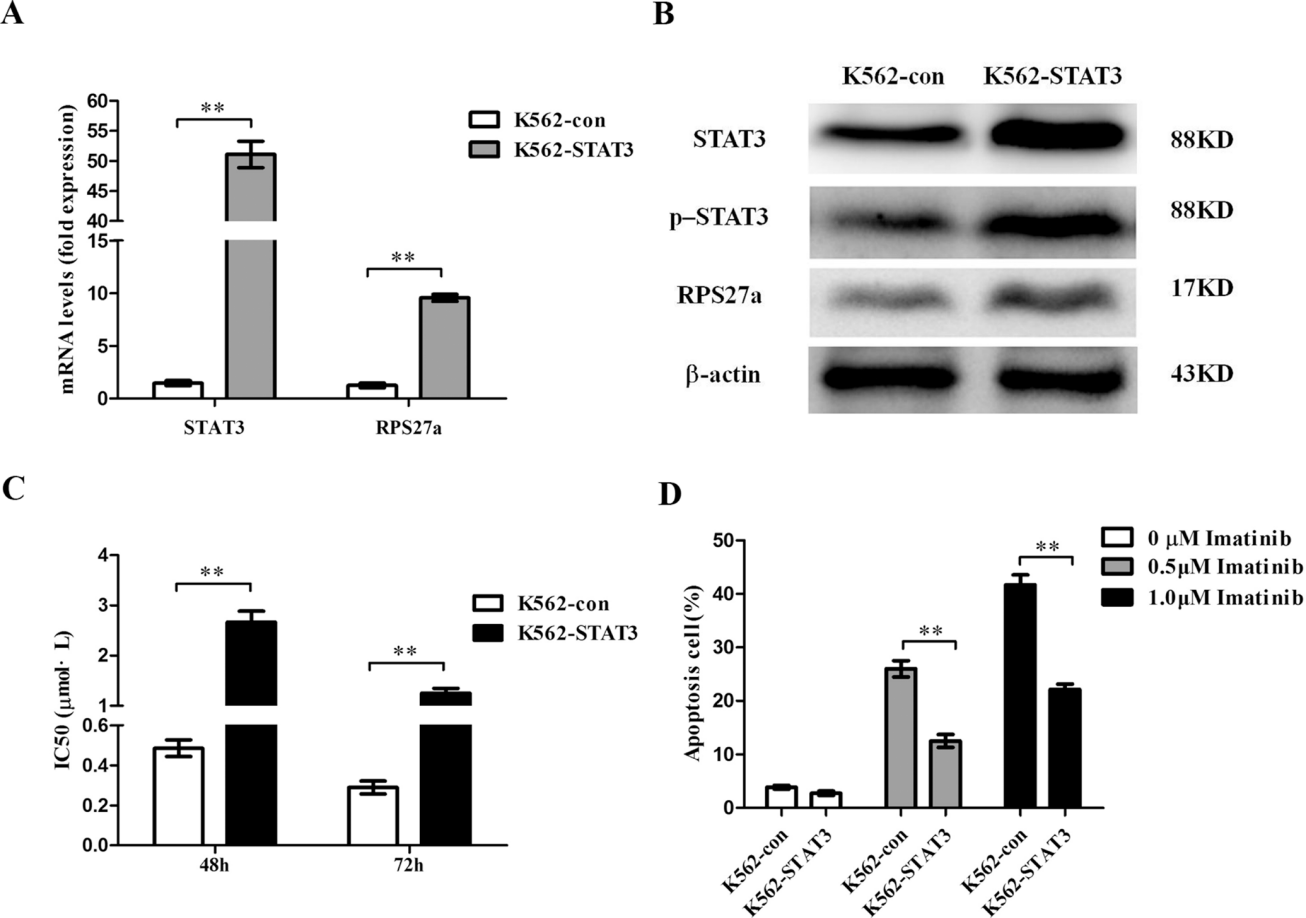
Validation of STAT3 overexpression and its effect on apoptosis in K562 cells (**A**) Relative STAT3 and RPS27a mRNA expression was assessed in K562 cells transfected with pcDNA3.1-STAT3 plasmids by qRT-PCR. (**B**) Lysates of K562 cells transfected with indicated plasmids were analyzed for STAT3, p-STAT3 and RPS27a protein by Western blot. (**C**) IC50s of K562-con and K562-STAT3 cells to imatinib at 48 h and 72 h was detected by MTT assays. (**D**) K562-con and K562-STAT3 cells were treated with or without 0.5 μM imatinib and 1.0 μM imatinib for 48 h and subjected to cell apoptosis analysis by flow cytometry analysis of Annexin-V labeling. Columns and bars represent mean from 3 parallel experiments and SD, respectively. *P < 0.05; **P < 0.01.

As STAT3 and RPS27a were highly expressed in CML-AP/BP and the expression of RPS27a could be up-regulated by p-STAT3, the role of STAT3 and RPS27a in CML cells was elucidated by using K562 cells. We investigated the level of RPS27a, p-STAT3 and STAT3 after infection with pcDNA3.1-STAT3. As expected, stable infection experiments showed RPS27a, p-STAT3 and STAT3 could be significantly up-regulated in K562-STAT3 cells in mRNA and protein levels, compared with those of K562-con cells (Figure 5A and 5B). Then we analyzed chemosensitivity of K562-STAT3 cells to imatinib treatment. As the expression of STAT3 was up-regulated in K562-STAT3 cells, MTT assays showed that the IC50s of imatinib at 48 h and 72 h increased (Figure 5C). The results indicated that overexpression of STAT3 could inhibit the sensitivity of K562 cells to imatinib treatment. Furthermore, we investigated whether the inhibited chemosensitivity could induce less K562-STAT3 cells undergoing apoptosis by using Annexin V-Alexa Fluor 647-A/PI double staining. The results showed that STAT3 overexpression alone didn't influence the percentage of apoptotic cells without imatinib treatment. Interestingly, STAT3 overexpression significantly decreased the percentage of apoptotic K562-STAT3 cells after incubation with 0.5 μM and 1.0 μM imatinib for 48 h compared with that of K562-con cells (Figure 5D).

**Figure 6:**
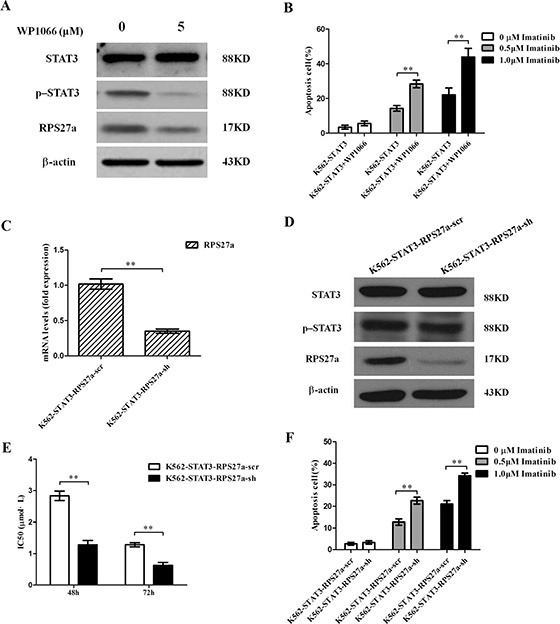
Validation of p-STAT3/RPS27a pathway blocking and its effect on apoptosis in K562-STAT3 cells (**A**) Lysates of K562-STAT3 cells incubated with or without WP1066 were analyzed for STAT3, p-STAT3 and RPS27a protein by Western blot. (**B**) K562-STAT3 cells were treated with or without 0.5 μM imatinib and 1.0 μM imatinib in the presence of 5 μM WP1066 for 48 h and subjected to cell apoptosis analysis by flow cytometry analysis of Annexin-V labeling. (**C**) Relative STAT3 and RPS27a mRNA expression was assessed in K562-STAT3 cells transfected with RPS27a shRNA plasmids by qRT-PCR. (**D**) Lysates of K562-STAT3-RPS27a-scr and K562-STAT3-RPS27a-sh cells were analyzed for STAT3, p-STAT3 and RPS27a protein by Western blot. (**E**) IC50s of K562-STAT3-RPS27a-scr and K562-STAT3-RPS27a-sh cells to imatinib at 48 h and 72 h was detected by MTT assays. (**F**) K562-STAT3-RPS27a-scr and K562-STAT3-RPS27a-sh cells were treated with or without 0.5 μM imatinib and 1.0 μM imatinib for 48 h and subjected to cell apoptosis analysis by flow cytometry analysis of Annexin-V labeling. Columns and bars represent mean from 3 parallel experiments and SD, respectively. *P < 0.05; **P < 0.01.

To confirm the effect of STAT3 overexpression in the regulation of cell apoptosis induced by imatinib of CML cells relies on the overexpression of p-STAT3. WP1066, one p-STAT3 inhibitor was applied to block the activity of p-STAT3 protein (Figure 6A). Then we analyzed chemosensitivity of K562-STAT3 cells to imatinib treatment. We investigated whether the enhanced chemosensitivity could induce more K562-STAT3 cells undergoing apoptosis by using Annexin V-Alexa Fluor 647-A/PI double staining in the presence of WP1066. As expected, inhibition of p-STAT3 protein by WP1066 significantly decreased the percentage of apoptotic K562-STAT3 cells after incubation with 0.5 μM and 1.0 μM imatinib for 48 h (Figure 6B). The results showed p-STAT3 inhibitor synergizes with imatinib in CML cells.

To confirm the effect of STAT3 overexpression in the regulation of cell apoptosis induced by imatinib of CML cells relies on the transactivation of RPS27a by p-STAT3. One RPS27a specific shRNA was applied to down-regulate RPS27a expression in K562-STAT3 cells. As expected, stable infection experiments showed RPS27a-shRNA could significantly down-regulate RPS27a expression in K562 -STAT3 cells in mRNA and protein levels (Figure 6C and 6D). Then we analyzed chemosensitivity of K562-STAT3-RPS27a-sh and K562-STAT3-RPS27a-scr cells to imatinib treatment. As the expression of RPS27a was down-regulated in K562-STAT3- RPS27a-sh cells, MTT assays showed that the IC50s of imatinib at 48 h and 72 h increased (Figure 6E). The results indicated that blocking the p-STAT3-RPS27a pathway could rescue the sensitivity of K562 cells to imatinib treatment. Then we investigated whether the enhanced chemosensitivity could induce more RPS27a-knockdown K562-STAT3 cells undergoing apoptosis by using Annexin V-Alexa Fluor 647-A/PI double staining. The results showed that blocking the p-STAT3/RPS27a pathway significantly increased the percentage of apoptotic K562-STAT3 cells after incubation with 0.5 μM and 1.0 μM imatinib for 48 h compared with that of K562-STAT3-RPS27a-scr cells (Figure 6F). The results showed the STAT3/p-STAT3/RPS27a pathway participated in the regulation of chemosensitivity of CML cells to imatinib treatment.

## DISCUSSION

In this study, we found that there was a striking increase of STAT3 and RPS27a mRNA expression in bone marrow samples from CML-AP/BP patients than that from CML-CP. And there was a positive correlation between the expression level of STAT3 and that of RPS27a. K562/G01 cells expressed significantly higher levels of STAT3 and RPS27a compared with those of K562 cells. RPS27a could be transactivated by p-STAT3 through the specific p-STAT3-binding sites located nt −633 to −625 and nt −486 to −478 in the RPS27a gene promoter in a dose-dependent manner. RPS27a transactivated by p-STAT3 could inhibit the percentage of apoptotic CML cells to imatinib.

RPS27a can perform extra-ribosomal functions. RPS27a are overexpressed in mouse liver cancer and some human tumors [34–36]. In previous study, we found that RPS27a was highly expressed in CML-AP/BP and imatinib resistant K562/G01 cells [31]. To explore the mechanism of RPS27a up-regulation in the transition of CML-CP to CML-AP/BP, We hypothesized that some highly expressed proteins in imatinib resistant CML cells contributed to the up-regulation of RPS27a gene. We found that STAT3 might transactivate RPS27a gene and there was a striking increase of STAT3 and RPS27a expression in bone marrow samples from CML-AP/BP patients than that from CML-CP. And there was a positive correlation between the expression level of STAT3 and that of RPS27a. Then we detected the levels of STAT3, p-STAT3 and RPS27a in K562 and K562/G01 cells. It was found that K562/G01 cells expressed significantly higher levels of STAT3, p-STAT3 and RPS27a compared with those of K562 cells. Then we investigated the changes of p-STAT3 and RPS27a expression after STAT3 was overexpressed in HEK293T cells. We found that there was a striking increase of p-STAT3 and RPS27a expression as STAT3 was overexpressed in HEK293T cells. It was implied that STAT3 could up-regulated the expression of p-STAT3 and RPS27a. While upregulation of RPS27a didn't occur in HEK293T cells when the phosphorylation of STAT3 was inhibited. It was implied that p-STAT3 played an indispensible role in the RPS27a up-regulation.

STAT3 plays a pivotal role in the hematopoietic system [18]. The level of p-STAT3 in imatinib resistant CML patients is higher than that in imatinib responsive ones [17]. Combined STAT3 and BCR-ABL1 inhibition induces synthetic lethality in therapy-resistant CML [16, 37]. In the study, we explored the possible relationship of p-STAT3 and RPS27a and their roles in CML cells. To this end, we generated a serial of RPS27a luciferase reporter gene constructs to analyze the trans-active effect of p-STAT3 on RPS27a. Luciferase transcriptional activity analysis showed that the luciferase activity of the S934B containing site nt −633 to −625 and nt −486 to −478) fragments, S594B containing site nt −486 to −478 fragment and S441P containing site nt −633 to −625 fragment were up-regulated by co-transfected pCMV5-STAT3 constructs. S934B luciferase activity was up-regulated in a dose-dependent manner. And the specificity of the DNA sequence was also verified by using point-mutated luciferase reporter construct Mu934B. To confirm the activation of RPS27a by p-STAT3. chromatin immunoprecipitation assays were performed using K562/G01 cells. The chromatin immunoprecipitation data demonstrated the *in vivo*-specific binding of p-STAT3 to promoter of the RPS27a gene. These data suggested that the STAT3/p-STAT3/RPS27a signal pathway might participate the transition from CML-CP to CML-BP.

In previous study, we found that RPS27a promotes inhibits the effect of imatinib on apoptosis of CML cell lines [31]. In the study, we investigated the regulation of STAT3/p-STAT3/RPS27a signal pathway on the effect of imatinib on apoptosis of K562 cells. We found that overexpression of STAT3 and p-STAT3 could inhibit the percentage of apoptotic k562 cells by activation of RPS27a and blocking the p-STAT3/RPS27a pathway significantly increased the percentage of apoptotic K562-STAT3 cells after incubation with imatinib. The results showed the STAT3/p-STAT3/RPS27a pathway participated in the regulation of chemosensitivity of CML cells to imatinib treatment.

In conclusion, STAT3 and RPS27a mRNA expression were up-regulated in the transition from CML-CP to CML-AP/BP. And there was a positive correlation between the expression level of STAT3 and that of RPS27a. RPS27a could be transactivated by p-STAT3 through the specific p-STAT3-binding site located nt −633 to −625 and −486 to −478 in the RPS27a gene promoter in a dose-dependent manner. RPS27a transactivated by p-STAT3 could inhibit chemosensitivity of CML cells to imatinib. It appears that drugs targeting STAT3/p-STAT3/RPS27a combining with TKI might represent a novel therapy strategy in some patients with TKI resistant CML.

## MATERIALS AND METHODS

### Patients

Bone marrow samples were obtained from 20 CML-CP patients and 15 CML-AP/BP enrolled in Department of Hematology, Shanghai Tenth People's Hospital, Tongji University School of Medicine. Two bone marrow samples were obtained from respective CP and BP of two CML patients (One patient: 38-year-old, male, progressed to AML. The other one: 45-year-old, female, progressed to AML). All samples were collected under informed consent of the subjects. The diagnosis and leukemia classification were based on 2008 World Health Organization criteria.

### Cell culture

K562, HEK293T, HEK293 and K562/G01 cell lines were maintained in our laboratory. The HEK293T and HEK293 cell lines were cultivated in Dulbecco's modified eagle medium and K562 and K562/G01 cell lines in RPMI 1640 medium containing 10% fetal bovine serum (Gibco, USA), 100 U/ml penicillin and 100 μg/ml streptomycin at 37°C in a humidified atmosphere of 5% CO_2_ in air. Imatinib at concentration of 1 μM was added to the culture system of K562/G01 cells to maintain resistance activity.

### RNA isolation and real-time quantitative PCR (qRT-PCR)

Total RNA was extracted using Trizol Reagent treated with DNase I and 2 μg RNA was reverse-transcribed using Superscript II RT following the manufacturer's instructions (Life technologies, USA). Primers for qRT-PCR were designed using Primer premier software 5.0. Human GAPDH primers used as an internal control were 5′-GAAGGTGAAGGTCGGAGTC-3′ (forward) and 5′-GAAGATGGTGATGGGATTTC-3′ (reverse). Human RPS27a primers were 5′-AGAAGAAGTCTTAC ACCACTCCC-3′ (forward) and 5′-TGCCATAAACACCC CAGC-3′ (reverse). Human STAT3 primers were 5′-CAGT TTCTTCAGAGCAGGTA-3′ (forward) and 5′-CTTGACT CTTGAGGGTTTT-3′ (reverse). The qRT-PCR products were 226 bp, 158 bp and 286 bp, respectively. The qRT-PCR was performed with SYBR Green PCR kit (Takara, Japan) following the manufacturer's instructions on the ABI PRISM7500 real-time PCR system. Thermal cycling conditions were 95°C for 5 min, followed by 40 cycles of 5 s at 95°C, and 34 s at 60°C. The qRT-PCR reactions were performed in a total volume of 20 μl, containing 2 μl of sample cDNA, 0.2 μM of each primer.

### Western blot

Total protein was extracted using RIPA lysis buffer (50 mM Tris-HCl, pH 7.5, 150 mM NaCl, 1% NP-40, 0.25% Na-desoxycholate, 5 mM EDTA, 1 mM NaF, 25 mM Na_3_VO_4_, 0.1 mM PMSF and 2 mg/ml Aprotinin). Protein concentration was determined by the BCA assay (Solarbio, China). The whole-cell lysates were heat-denatured at 95°C for 5 min before run on 10% SDS- PAGE. After SDS-PAGE, the proteins were electro-transferred onto nitrocellulose membranes, blotted with each primary antibody, incubated in secondary antibody and then detected with enhanced chemi-luminescence detection reagent (Pierce, USA).

### Luciferase assay

The DNA sequence of 1.6-kb full-length human RPS27a gene (FL-RPS27a) was from nt −1644 to −7 of the promoter sequence of RPS27a. The DNA fragment was cloned from the total DNA of K562 cells by PCR. The primers were 5′-AGCCAAGATCTGCCAAGGTA-3′ (forward) and 5′-CACTCCTTAGTGTGACACGCTTT-3′ (reverse). After transfected by calcium phosphate-mediated precipitation for 48 h, HEK-293 cells were collected for assays of β-galactosidase and luciferase activity according to the Dual-Luciferase Reporter 1000 Assay System Technical Manual (Promega, Madison, WI, USA) by luminometer (Lumat LB 9507, Berthold Technologies GmbH & Co. KG, Baden-Württemberg, Germany). The following combinations of plasmids were used: 750 ng S934B, Mu934B, S594B or S441P reporter plasmids with different dosage of pCMV5-STAT3 and with 750 ng β-galactosidase plasmids.

### Chromatin immunoprecipitation analysis

Chromatin immunoprecipitation analysis was performed with sonication method. The purified chromatin from K562/G01 cells was immunoprecipitated with anti-p-STAT3 antibody (Abcam). Anti-STAT3 antibody (Abcam), immunoglobulin G isotype and H2O were used as control. The eluted DNA was purified, and used as PCR template to amplify the promoter sequences between nt −670 and −467 containing putative p-STAT3-binding site at nt −633 to −625 and nt −486 to −478 of RPS27a promoter with the following primers: 5′-GAGCGAAATTCCGTCTC-3′ (forward) and 5′-TGACATTCAGCCTCTGC-3′ (reverse). The PCR products were 184 bp.

### Short hairpin RNA-mediated RNA interference studies

The cDNA sequence of RPS27a was obtained from GenBank (NM_002954.5). A RPS27a-specific targeting sequence was designed with the software from Ambion. The target sequence for RPS27a was 5′-TTAGTCGCCTTCGTCGAGA-3′. The shRNA expressing plasmid specifically targeting RPS27a was cloned into pLKO-1 vector to obtain pLKO-shRNA construct. A scramble shRNA sequence (AATAGACTCAGCAAATGCG) with no homology to any human gene was used as a negative control (scr). For the production of lentivirus, HEK293T cells were co-transfected with pLKO-shRNA or pLKO-scr and pCMV, pMDG by Ca_3_(PO_4_)_2_ precipitation. Lentivirus supernatants were harvested 48 h after transfection, and used to infect K562-STAT3 cells (termed as K562-STAT3-RPS27a-sh and K562-STAT3-RPS27a-scr). Infected cells were selected by puromycin 7 days after infection for at least 2 weeks to obtain stable clones. Inhibition of RPS27a expression was measured by qRT-PCR as well as by Western blot using a rabbit anti-RPS27a monoclonal antibody (abcam, UK).

### 3-(4, 5-Dimethyl-2-thiazolyl)-2, 5-diphenyl-2H-tetrazolium bromide (MTT) assays

5 × 10^3^ cells per well were seeded into 96-well plates in 100 μl volume and grown in RPMI1640 medium supplemented with 10% fetal bovine serum at 37°C. Cells were cultured for 48 h and 72 h, respectively. After indicated time of incubation, 10 μl of MTT reagent was added. Then the cells were incubated at 37°C for an additional 4 h, and 100 μl of 10% sodium dodecyl sulfate-HCl 0.1 N was added to each well. The mixture was incubated at 37°C overnight and then the absorbance of formazan product was measured by Versa Max tunable microplate reader (Molecular Devices, USA) at the wavelength of 546 nm. The *in vitro* cytotoxicity of K562 and K562-STAT3 cells and their transfectants were assessed by MTT assays.

### Apoptosis analysis by flow cytometric assay

Phosphatidylserine externalization was analyzed with Annexin V-Alexa Fluor 647-A/PI Apoptosis Analysis Kit by a FACS Calibur flow cytometer (BD, USA) for cell apoptosis. Apoptosis was quantified as the percentage of Annexin V positive cells.

### Statistical analysis

All experiments were conducted at least three times and data were presented as mean ± SD. Statistical analysis was performed with the SPSS software package (version 17.0; SPSS). *P* < 0.05 was deemed statistically significant.
